# Histone acetylation: novel target for the treatment of acute lymphoblastic leukemia

**DOI:** 10.1186/s13148-015-0151-8

**Published:** 2015-11-04

**Authors:** Cheng Zhang, Jiang F. Zhong, Andres Stucky, Xue-Lian Chen, Michael F. Press, Xi Zhang

**Affiliations:** Department of Hematology, Xinqiao Hospital, Third Military Medical University, Chongqing, 400037 People’s Republic of China; Department of Diagnostic Sciences & Biomedical Sciences, Ostrow School of Dentistry, University of Southern California, Los Angeles, CA 90033 USA; Department of Pediatric, Keck School of Medicine, University of Southern California, Los Angeles, CA 90033 USA; Department of Pathology, Keck School of Medicine, University of Southern California, Los Angeles, CA 90033 USA

**Keywords:** Histone acetylation, Acute lymphoblastic leukemia, Histone acetyltransferase, Histone deacetylase, Biomarker, Clinical application

## Abstract

Acute lymphoblastic leukemia (ALL) has been generally considered a genetic disease (disorder) with an aggressive tumor entity of highly proliferative malignant lymphoid cells. However, in recent years, significant advances have been made in the elucidation of the ALL-associated processes. Thus, we understand that histone acetylation is involved in the permanent changes of gene expression controlling ALL developmental outcomes. In this article, we will focus on histone acetylation associated with ALL, their implications as biomarkers for prognostic, and their preclinical and clinical applications.

## Background

Acute lymphoblastic leukemia (ALL) is an aggressive tumor entity of highly proliferative malignant lymphoid cells. This leukemia subtype occurs most frequently in children with incidence peaks between 2 and 5 years of age and is one of the most common childhood malignancies worldwide [1]. Survival in ALL has improved in clinical trials with treatment modification based on patients’ pharmacodynamics and pharmacogenomics. However, prognosis remains poor in infants and adults, and innovative approaches are needed to further improve survival while reducing adverse effects. Almost all human cancer types contain epigenetic alterations that contribute to cancer development due to the regulatory role during transcription of epigenetic modifications in genes [2]. Disturbance of correct epigenetic configuration is postulated to act as a first seminal event in carcinogenesis leading to early abnormal clonal expansion of stem/progenitor cells [2, 3].

The aberrant histone acetylation is involved in the permanent changes of gene expression controlling ALL phenotype. Moreover, since histone acetylation can be reversed, the development of drug-based treatments for targeting proteins and enzymes involved in the regulation of histone acetylation in ALL has become an attractive therapeutic strategy. In this article, we will focus on the histone acetylation associated with ALL, their implications as biomarkers for prognostic, and their preclinical and clinical applications.

## Review

### Histone acetylation

Histone acetylation and deacetylation, essential parts of gene regulation, are the processes through which the lysine residues within the N-terminal tail protruding from the histone core of the nucleosome are acetylated and deacetylated as part of gene regulation.

These reactions are typically catalyzed by enzymes with histone acetyltransferase (HAT) or histone deacetylase (HDAC) activity. The major HATs are GNAT family, MYST family, and CBP/p300 family [3, 4]. The HDACs with four classes (classes I, II, III, and IV) include HDAC1–11 and Sirtuins [5, 6]. Acetylation is the process that an acetyl functional group is transferred from one molecule to another. Deacetylation is simply the reverse reaction that an acetyl group is removed from a molecule. Acetylated histones of octameric proteins that organize chormatin into nucleosomes and ultimately higher order structures represent a type of epigenetic marker within chromatin. Acetylation removes the positive charge on the histones, thereby decreasing the interaction of the N termini of histones with the negatively charged phosphate groups of DNA. As a consequence, the condensed chromatin is transformed into a more relaxed structure that is associated with greater levels of gene transcription; however, this relaxation can be reversed by HDAC activity [7].

### Role of histone acetylation

**Fig. 1:**
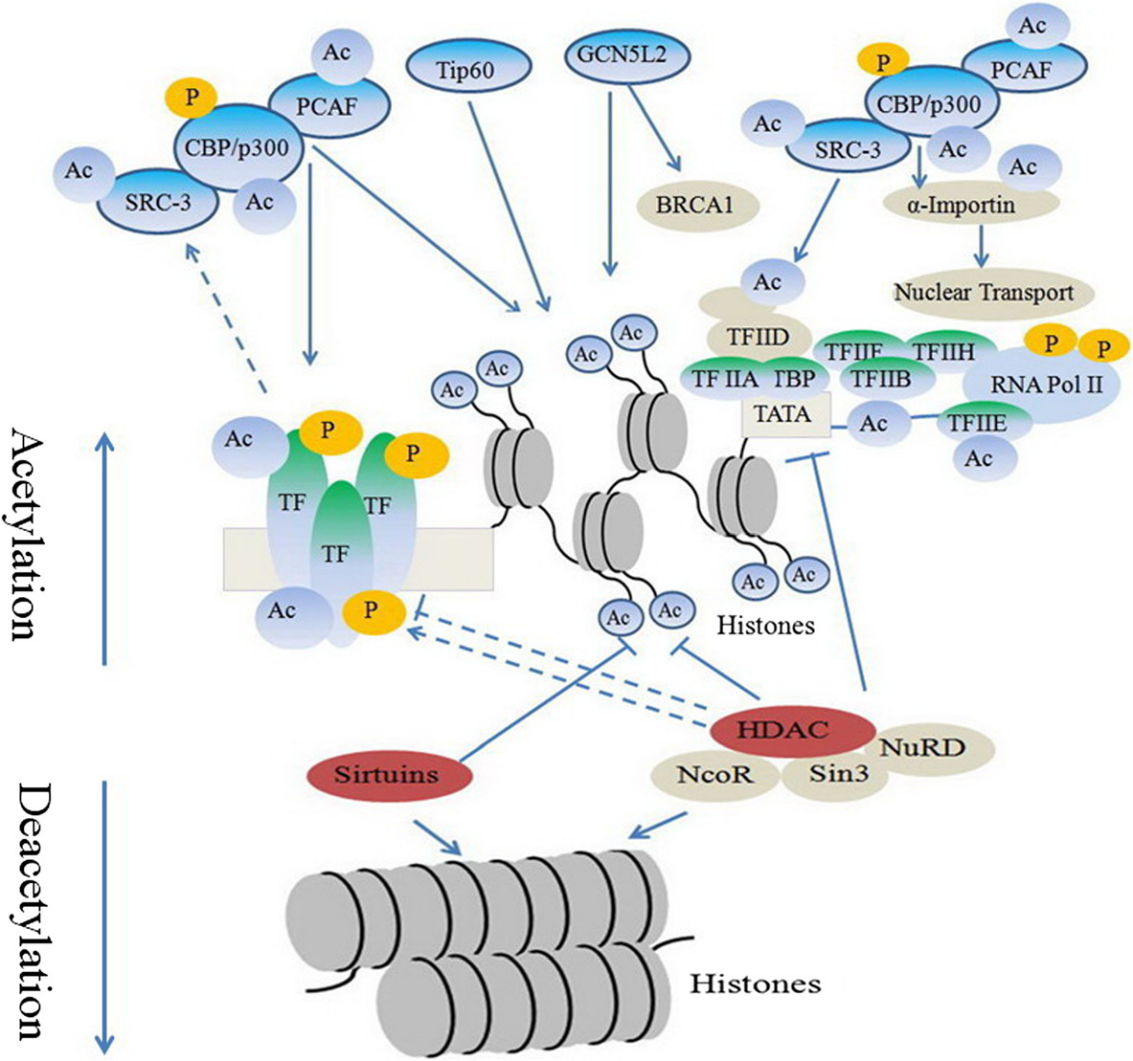
Histone acetylation alters chromatin structure. Acetylation removes the positive charge on the histones with histone acetyltransferases, which is referred to as euchromatin. As a consequence, the condensed chromatin is transformed into a more relaxed structure that is associated with greater levels of gene transcription. However, this relaxation can be reversed by histone deacetylase, which is referred to as heterochromatin. Acetylation has been closely associated with increases in transcriptional activation while deacetylation has been linked with transcriptional deactivation

Histone acetylation has been closely associated with increases in transcriptional activation while deacetylation has been linked with transcriptional deactivation and is known as gene silencing, and histone acetylation also causes changes in transcription activity (Fig. 1) [8–15]. Histone proteins modified by acetyl groups add negative charges to positive lysines, thus, reducing the interaction between DNA and histones [16–19]. This opens up the usually tightly packed nucleosome and allows transcription machinery to come into contact with the DNA template, leading to gene transcription. The acetyl group is removed by one of the HDAC enzymes during deacetylation, allowing histones to interact with DNA more tightly to form compacted nucleosome assemblies, which can effectively silence gene transcription.

Another implication of histone acetylation is to provide a platform for protein binding. As a posttranslational modification, acetylation of histones can attract proteins to elongated chromatin that has been marked by acetyl groups. Therefore, acetyl mark provides a site for protein recognition where transcription factors interact with the acetylated histone tails via their bromodomain [20].

Patterns of posttranslational modifications on histones, collectively, can direct specific cellular functions [21]. The specific addition of single or multiple modifications on histone cores can be interpreted by transcription factors complexes, leading to functional implications [22]. Acetylation patterns of H4 histones suggested that these modification patterns are collectively maintained during mitosis and meiosis leading to long-term changes in gene expression. Acetylation patterns are transmitted and interconnected with protein binding ability and functions in subsequent cell generation. Specific histone site acetylation has a regulatory role in gene transcriptional activation [23].

Acetylated histones represent a type of epigenetic marker within chromatin. Modifications of histones cannot only cause secondary structural changes at their specific points but can also cause structural changes in distant locations which can also affect function. As the chromosome is replicated, modifications on the parental chromosomes are handed down to daughter chromosomes. It has been shown that, expression of genes may still be effected even past one replication and in fact has been observed many cell generations later.

### Altered histone acetylation enzyme

#### HATs

CREBBP (also called CBP) or EP300 (also called p300) have been found fused to MLL, MOZ, and MORF. Interestingly, MOZ-CBP, a fusion protein associated with AML carrying the t(8;16)(p11, p13) translocation, is composed of two proteins with acetylating activities: CBP and MOZ, the latter belonging to the MYST family of acetyltransferases [24]. The different fusion proteins described contribute to leukemic transformation most likely by a mechanism involving mistargeted histone acetylation and thus aberrant activation of gene expression [25]. In 84 % (73/86) of ALL patients, *CREB* is overexpressed at diagnosis but not in remission nor in non-leukemia samples [26]. By contrast, the parallel expression of the cAMP early inducible repressor (*ICER*), which represses CREB activity by competing for the CREB consensus site, appears downregulated at diagnosis but neither in remission nor in control samples [26]. Thus, it is plausible that *CREB* overexpression leads to target gene upregulation and increased cell proliferation and survival that are not counteracted by the insufficient level of *ICER* expression. Despite the apparently good prognosis, 15 % of high hyper-diploid (HD) childhood ALL cases relapse and the majority of these cases have mutations in the CREBBP HAT domain [27, 28]. Relapse of ALL is a leading cause of death due to disease in young people, but the reasons for poor prognosis are still to be elucidated. Mullighan and colleagues performed targeted resequencing of 300 genes in 23 matched relapse-diagnosis B-ALL pairs. Genome-wide profiling of structural DNA alterations in ALL identified multiple sub-microscopic somatic mutations targeting key cellular pathways and demonstrated evolution in genetic alterations from diagnosis to relapse [29]. Many of the mutations that have been identified concern the transcriptional co-activators *CREBBP* and *NCOR1*, the transcription factors *ERG*, *SPI1*, *TCF4*, and *TCF7L2*, components of the Ras signaling pathway, histone genes, genes involved in histone modification (*CREBBP* and *CTCF*), and genes target of DNA copy number alterations [29]. The parallel analysis of an extended cohort of diagnosis-relapsed cases and acute leukemia cases that did not relapse showed that the 18.3 % relapsed cases had sequence deletion or mutations in *CREBBP* [27, 29]. *CREBBP* is expressed in leukemia cells and normal B-cell progenitors, and the mutant *CREBBP* alleles are expressed in ALL cell lines harboring mutations. Mutations at diagnosis or acquired at relapse consist in truncated alleles or deleterious substitutions in conserved residues of the HATs domain, impairing histone acetylation and transcriptional regulation of *CREBBP* targets, including glucocorticoid responsive genes. In mice, the homozygous deletion of *CREBBP* or *EP300* is lethal due to developmental abnormalities whereas *CREBBP*(+/−) mice show defects in B lymphoid development and an increased incidence of hematopoietic tumors [30]. Both *CREBBP* and *EP300* sequence mutations have been reported in solid tumors and, more recently, also in hematological malignancies, whereas rare *EP300* mutations have been detected in some ALL cell lines [29, 31]. Many identified mutations are related to transcriptional and epigenetic regulation in ALL treatment resistance. It is worth outlining that the high incidence of *CREBBP* mutations found in relapse-prone HD ALL cases discloses the possibility of a targeted customized treatment in this genetic subgroup [28]. Recently, higher expression levels of *KAT7*, *KAT2A*, *KAT6B*, and *CSRP2BP* were found in B-ALL; however, the functional role of this overexpression in leukemogenesis is unknown. Notably, it was demonstrated that KAT2A acetylates the E2A-PBX1 oncoprotein (resulting from the fusion of *TCF3*-*PBX1* genes), increasing its stability in B-ALL cells [32].

Histone acetylations are not only restricted to B-ALL but also are a notable feature of T-ALL, particularly the aggressive subtype early T cell precursor (ETP) ALL. Whole genome sequencing of 12 cases of ETP ALL identified mutations in genes encoding components of the polycomb repressor complex 2 (PRC2), including deletions and sequence mutations of *EZH2*, *SUZ12*, and *EED* [33]. Loss of function mutations and deletions of *EZH2* and *SUZ12* genes have also been found in T-ALL, where authors implicate the tumor suppressor role of the PRC2 complex [34]. CREB activation can also have an important role in the complex cross talk among pro- and anti-apoptotic pathways in Jurkat T cells [35].

#### HDACs

Changes in histone acetylation can contribute to carcinogenesis through altered transcriptional regulation of genes involved in various biological processes, such as cell cycle regulation differentiation, apoptosis, cell adhesion, and angiogenesis. Especially, increased expression of HDACs, leading to reduced histone acetylation, is known to be widespread among cancers. Moreno et al. identified higher expression of several *HDAC* genes (i.e., *HDAC2*, *HDAC3*, *HDAC8*, *HDAC6*, and *HDAC7*) in ALL when compared with normal bone marrow. Furthermore, *HDAC6* and *HDAC9* were upregulated in B cell ALL, whereas *HDAC1* and *HDAC4* were overexpressed in T cell ALL [36]. In addition, Tao et al. recently confirmed that *HDAC2* was overexpressed in ALL [37]. Moreover, increased expression of *HDAC3*, *HDAC7*, and *HDAC9* has been associated with poor prognosis in childhood ALL, and cells from these patients were found to display increased HDAC activity [36, 38]. H4 acetylation was recently suggested as a prognostic marker in new ALL patients, as well as in patients at first relapse. Indeed, high levels of H4 acetylation were correlated with an increased overall survival, although the authors stated that the study has to be confirmed on a greater number of patients and adding the analysis of H3 acetylation levels [39, 40].

Gruhn and colleagues also identified the relevance of HDACs for childhood ALL. In this experiment, the expression of HDAC1–11 was determined in samples from 93 patients with primary ALL and eight healthy donors. They found that HDAC1, HDAC2, and HDAC8 expression was significantly higher in ALL samples. High expression of HDAC4 was associated with a high initial leukocyte count, T cell ALL, and poor response to prednisone. These data show that HDAC4 could be a drug target in childhood ALL, especially in those responding poorly to prednisone [41].

In addition to the discovery of somatic mutations in epigenetic machinery in ALL, messenger RNA (mRNA) expression of HDACs has also been shown to be dysregulated. Moreno and colleagues presented an analysis of the mRNA levels of 12 different HDAC isoforms in childhood ALL. HDAC2, HDAC3, and HDAC6 to HDAC8 mRNAs were overexpressed in ALL compared with normal bone marrow samples. HDAC1 and HDAC4 levels were also found to be higher in T cell ALL, whereas HDAC5 mRNA was higher in B cell ALL. This study investigates the correlation of HDAC transcript levels with patient’s survival resulting in the association of overexpression of HDAC3, HDAC7 and HDAC9 with poor prognosis [36]. The analysis of HDAC mRNAs from ALL samples revealed an overexpression of HDAC6 and SIRT1 and a downregulation of HDAC5 [42]. Given the compelling evidence of HDAC’s involvement in tumor development and progression, inhibitors of HDACs have emerged as an attractive therapeutic option for hematologic malignancies. Altogether, aberrant histone acetylations have been associated with disease progression and relapse of ALL and provided a molecular basis for the pharmacological use of HDAC inhibitors (HDACis) in ALL treatment.

### Histone acetylation in preclinical and clinical trials

Along with the fact that unfavorable epigenetic alterations might be reversible, the indisputable role of epigenetics in cancer has favored the development of novel epigenetic drugs. There are 377 known epigenetic proteins according to the Structural Genomics Consortium. In recent years, several small-molecule inhibitors have been developed to target epigenetic regulators, including various HMTs (e.g., EZH2, EZH1, DOT1L, and SUV39H1), HDMs (e.g., LSD1, LSD2, and JMJD2) and a histone acetyltransferase (p300/CBP). However, epigenetic-based studies have only yielded approval for drugs affecting two classes of epigenetic regulators, DNA methyltransferases and HDACs.

#### HAT inhibitors

Only a few molecules have been brought to light as HAT inhibitors, which have not been extensively studied in leukemia [43]. Anacardic acid, garcinol, and curcumin have been proven to be valid natural HAT inhibitors. The first synthetic compounds designed were coenzyme A (CoA)-conjugated peptide analogs (e.g., LysCoA, H3-CoA-20, and others). Subsequently, other compounds such as garcinol analogs, the g-butyrolactone MB-3, isothiazolones, and quinoline derivatives have been reported to inhibit specific HAT members and to be effective in blocking proliferation of some solid tumor cell lines, but the mechanism of action is still not elucidated. A p300-specific molecule, the pyrazolone-furan C646, is able to suppress proliferation of melanoma and lung cancer cell lines, reinforcing the idea that HATs are promising targets for cancer therapy, including leukemia.

#### HDAC inhibitor

**Fig. 2:**
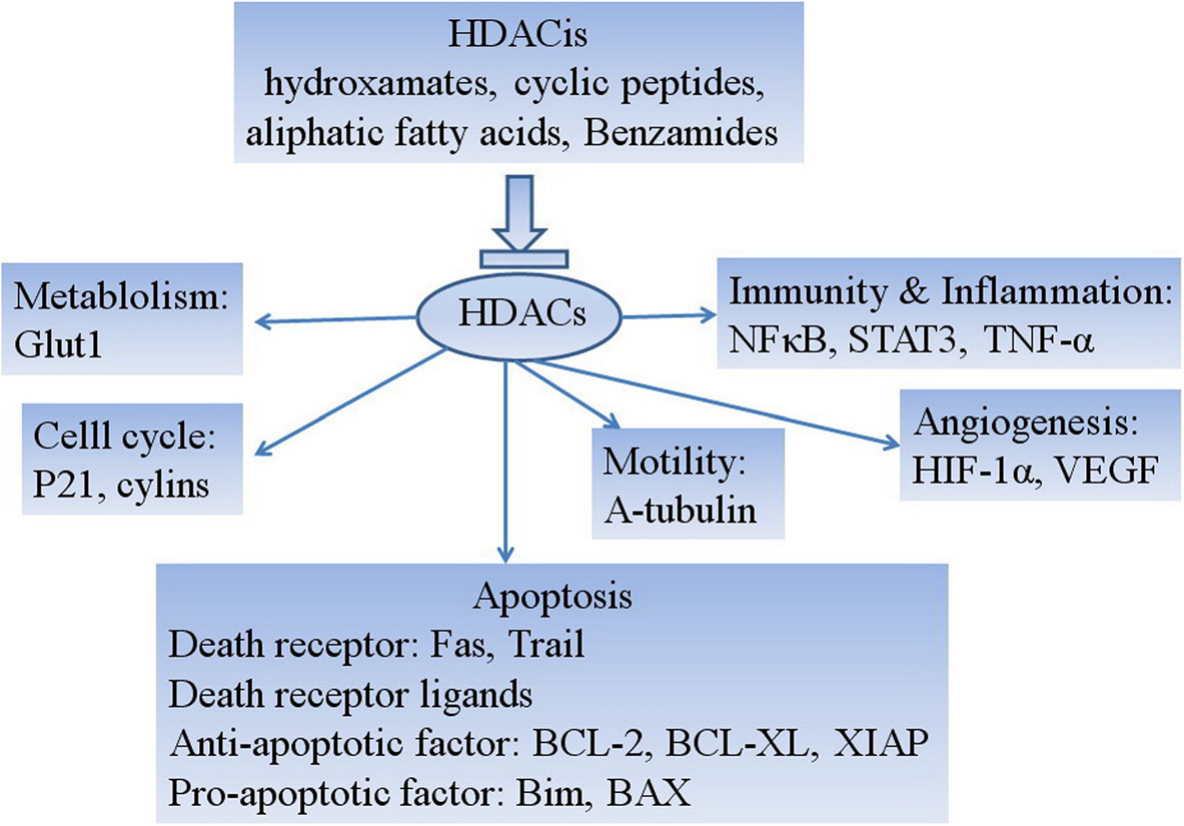
Major mechanism of histone deacetylase inhibitors action. Enhanced histone acetylation in a variety of tumors results in modification of expression of the genes involved in cell signaling, which involved in several biological processes such as cell cycle arrest and apoptosis induction and metabolism and angiogenesis

HDACs are a powerful new class of anticancer agents that have the potential to restore normal histone acetylation status of cells in order to enhance gene transcription. Different HDACis induce death of cancer cells by different mechanisms that include changes in gene expressions and alterations of both histone and non-histone proteins. Enhanced histone acetylation in a variety of tumors results in modification of expression of the genes involved in cell signaling, which involved in several biological processes such as cell cycle arrest and apoptosis induction and metabolism and angiogenesis (Fig. 2) [44–48]. Many molecules with HDAC-inhibiting activities have been discovered in the last few years. Newer compounds have been studied in the clinic with varying results, such as panobinostat (LBH589), givinostat (ITF2357), mocetinostat (MGCD01030), belinostat (PXD101), pracinostat (SB939), and entinostat (MS275), the class I-specific agents CHR-3966, chidamide (CS055/HBI-8000), class I- and class II-specific AR-42, hydroxamides quisinostat (JNJ-26481585), and abexinostat (PCI-24781) [49, 50]. These inhibitors affect multiple cellular processes and have been shown to induce differentiation, cell cycle arrest and apoptosis. Moreover, they have been used to inhibit cell migration, invasion, and angiogenesis in cancer cell lines [51]. The potential importance of these changes is highlighted by the promising activity of several other drugs from the same class that target epigenetic alterations [52]. Some of which have entered clinical trials involving hematological or solid tumors, either as monotherapies or in combination with other drugs [49, 53].

HDACis may be both specific against only some HDACs (HDAC isoform-selective inhibitors) or against all types of HDACs (pan-inhibitors). They can be classified into four groups according to their chemical structure: hydroxamic acids, aliphatic acids, benzamides, and cyclic tetrapeptides (Table 1) [53–55]. Hydroxamic acids include trichostatin A, vorinostat, CBHA, LAQ-824, PDX-101 (belinostat), LBH-589 (panobinostat), ITF-2357, and PCI-24781 [56]. Cyclin tetrapeptides include romidepsin (depsipeptide, FK228, FR901228), apicidin, and trapoxin A and B [57, 58]. The aliphatic acids include valproic acid (VPA), butyric acid, AN-9 (pivanex), and phenylbutyric acid [59]. Benzamides include entinostat [SNDX-275, MS-275 (entinostat)] and mocetinostat (MGCD0103) [60]. However, only a few HDACis were studied and/or approved by the FDA for the treatment of leukemia (Table 2). These HDACis in preclinical and clinical trials for ALL were reported as follow.

**Table 1:** HDACis under clinical development

Chemical structure	Name	Target	Study phase
Hydroxamates	SAHA (vorinostat)	Pan-inhibitor	Phase III alone or in combination
	PXD101 (belinostat)	Pan-inhibitor	Phase II alone or in combination
	LBH589 (panobinostat)	Pan-inhibitor	Phase II alone or in combination
	ITF2357 (givinostat)	Pan-inhibitor	Phase II alone or in combination
	4SC-201 (resminostat)	Pan-inhibitor	Phase II alone or in combination
	PCI 24781 (abexinostat)	Classes I and II	Phase II alone or in combination
Cyclic peptides	Depsipeptide/FK228 (romidepsin)	Classes I	phase III alone or in combination
Aliphatic fatty acids	Valproic acid	Classes I and IIa	Phase II alone or in combination
	Butyrate	Classes I and IIa	Phase II alone or in combination
Benzamides	MS-275 (entinostat)	Class I	Phase II alone or in combination
	MGCD0103 (mocetinostat)	Class I/IV	Phase II alone or in combination

*HDACis* HDAC inhibitors

**Table 2:** HDACis in clinical trials for leukemia

Chemical structure	Name	Target	Study phase
Hydroxamates	SAHA (vorinostat)	Pan-inhibitor	Phase I/II alone or in combination
	PXD101 (belinostat)	Pan-inhibitor	Phase I/II alone or in combination
	LBH589 (panobinostat)	Pan-inhibitor	Phase I/II alone or in combination
Cyclic peptides	Depsipeptide/FK228 (romidepsin)	Classes I	Phase I/II alone or in combination
Aliphatic fatty acids	Valproic acid	Classes I and IIa	Phase II alone or in combination
	Butyrate	Pan-inhibitor	Phase I/II alone or in combination
Benzamides	MS-275 (entinostat)	Class I	Phase I alone or in combination
	MGCD0103 (mocetinostat)	Class I/IV	Phase I alone or in combination

*HDACis* HDAC inhibitors

#### Vorinostat

Vorinostat, also called suberoylanilide hydroxamic acid (SAHA), blocks the enzymatic activity of both Class I (HDAC1, HDAC2, and HDAC3) and Class II (HDAC6) HDACs at low nanomolar concentrations (IC50 < 86 nM) by directly binding to the catalytic site of these enzymes, and its therapeutic properties were first demonstrated in nude mice transplanted with human prostate tumors for its anticancer potential [61, 62]. Recently, vorinostat has been approved by the US Food and Drug Administration for the treatment of cutaneous T cell lymphoma and along with Panobinostat (LBH589), and Belinostat (PXD101), they are undergoing clinical investigation for leukemia [63, 64].

Vorinostat has numerous effects on biological processes including cell cycle progression, apoptosis, and differentiation at the cellular level, as well as angiogenesis inhibition and immune response modulation at the tissue level [61]. One model for the antitumor action of SAHA is that its inhibition of HDAC activity, and subsequent accumulation of acetylated histones, leads to the activation of genes whose expression causes induction of differentiation or apoptosis, thus inhibiting tumor growth, which is based on the finding that the expression of a relatively small number of genes (2–10 % of expressed genes) is regulated following exposure of tumor cells to vorinostat [61]. One of the most commonly induced genes is the cyclin-dependent kinase inhibitor p21. However, rather than promoting apoptosis in tumor cells, it appears that vorinostat induced expression of p21 causes a cell cycle arrest in a p53-independent manner [65]. Moreover, increased acetylation of transcription factors such as p53, HIF-1α, and E2F and increased acetylation of cytoplasmic proteins such as α-tubulin, HSP90 and cortactin also contribute to vorinostat-induced cell cycle arrest, induction of cell death, and the inhibition of tumor growth [61].

Using two NOD/SCID mouse models, the efficacy of vorinostat was observed in B cell precursors childhood ALL in vivo. In fact, vorinostat was administered in a dose of 50, 100, or 150 mg/kg for 21 days, in both subcutaneous and intravenous models, and caused a clear growth suppression of the xenograft tumors [66].

Vorinostat not only modulated the gene expression signature characteristic of relapse in ALL cell lines and patient samples but also showed a synergistic effect when given sequentially with chemotherapy. Coadministration of vorinostat plus the highly effective antileukemic drug methotrexate (MTX) can synergize to induce apoptotic death in ALL cells. When used as single agents, both MTX and vorinostat can induce growth arrest, cell death, and apoptosis. However, MTX plus vorinostat synergistically increase apoptotic cell death and decrease viability in all B-precursor-ALL (SupB15, REH, NALM6, RCH-ACV) and T-ALL (CCRF-CEM) cell models tested [67]. Synergistic effect of vorinostat with dexamethasone in both in vitro and in vivo models for glucocorticoid resistance is associated with epigenetic silencing of the *BIM* gene in pediatric ALL [68]. A trial studying the efficacy of the use of decitabine and vorinostat together with combination chemotherapy in treating patients with relapsed/refractory ALL or lymphoblastic lymphoma with 2 to 60 years of the age was performed [69]. Another pilot study was being performed using vorinostat and decitabine before and during chemotherapy with vincristine, dexamethasone, mitoxantrone, and peg-asparaginase in pediatric patients with relapsed ALL [70].

Numerous in vitro and in vivo studies have already demonstrated the antileukemic activity of HDACis in ALL, either alone or in combination with bortezomib (proteasome inhibitor), decitabine (DNMT inhibitor), MK-0457 (Aurora kinase inhibitor), KW-2449 (BCR-ABL1 tyrosine kinase inhibitor), or standard chemotherapy [66, 71–77]. However, vorinostat was evaluated in pediatric preclinical trials and was not effective as a single agent [78]. The mechanism of action of LBH589 in two Ph^−^ ALL cell lines (T cell MOLT-4 and preB cell Reh) was investigated. Low nanomolar concentrations of LBH589 induced cell cycle arrest, apoptosis, and histone hyperacetylation. LBH589 treatment also increased mRNA levels of proapoptosis, growth arrest, and DNA damage repair genes [74]. Despite overwhelming preclinical data, very few phase I clinical trials have been conducted to assess HDACis in ALL and existing studies involved only a handful of ALL patients. Specifically, a phase I study of LBH589 included one patient with ALL, while a phase I study of vorinostat included only two ALL patients [79, 80]. The study found that intravenous administration of LBH589 was well tolerated at doses <11.5 mg/m2, while higher doses induced cardiac and other toxicities like nausea, diarrhea, vomiting, hypokalemia, loss of appetite, and thrombocytopenia [79]. Therefore, there are insufficient clinical data to establish whether this class of drugs will be efficacious in treating ALL. However, HDACis remain as promising candidates for combination therapies involving conventional chemotherapy or other types of inhibitors [81, 82].

#### Aliphatic fatty acids

Some compounds of natural origin have been initially isolated for their ability of inhibiting deacetylation. Sodium butyrate and trichostatin A are the prototypes of two families of inhibitors, chemically classified as short-chain fatty acids and hydroxamic acids, respectively. Trichostatin A can induce apoptosis of ALL cell lines including Sup-B15, TMD-5, SEM, and NALM-6 [83]. Sodium butyrate exhibits a short half-life that limits its therapeutic application; however, from its structure, other more promising molecules have been developed, in particular, sodium phenylbutyrate and AN-9 (Pivanex), which demonstrated selective toxicity for leukemia cells against healthy blood cells [84, 85]. AN-9 is a relatively new member of an established family of acyloxyalkyl ester prodrugs of carboxylic acids that undergo rapid hydrolysis, and its anticancer effect is assumed to stem primarily from the release of butyric acid [83]. AN-9 has antiproliferative and cytotoxic effects on a doxorubicin-resistant T-ALL and a relapsed infant ALL characterized by an 11q23 rearrangement and a very poor prognosis. The mechanism behind the antiproliferative effect of AN-9 appeared to be generally p21-independent, and the increased apoptosis was thought to be mediated through the reduction in the expression of antiapoptotic bcl2 gene or alternatively through the induction of genes involved in the death receptor pathway [85]. At present, no clinical trial was studied on the role of AN-9 on the treatment of ALL in clinic.

#### Valproic acid

Another well-known short-chain fatty acid is valproic acid (VPA), a derivative of valeric acid commonly used to treat epilepsy which demonstrates selective inhibition of class I and IIa HDACs [86]. Although VPA is a weak HDACis, its long-term availability as an antiepileptic drug prompted its evaluation on oncology as an epigenetic acting drug. VPA can inhibit the proliferation and increase the apoptosis of Jurkat T cells in concentration-dependent manner [87]. VPA also inhibits proliferation, and induces apoptosis and histone H4 hyperacetylation in B cell precursor-ALL cell lines (Reh, Nalm6, Z33) in vitro [76]. Moreover, the administration of VPA reduces the tumor growth significantly in two NOD/SCID mouse models of B cell precursor-ALL. The study showed that VPA treatment was able to inhibit the leukemia-induced splenomegaly of animals after intravenous challenge with ALL blasts, while no apparent toxicity was detected [74]. Several studies showed that VPA is more efficacious in combination with other agents [88, 89]. VPA can increase responsiveness of Philadelphia-positive cell lines to cytarabine [90]. Although phase I/II studies of VPA monotherapy or in combination have been completed in adult patients, the patients of these trials came from acute myeloid leukemia [91]. Many studies also suggested that VPA affects hematopoiesis, inducing anemia in epileptic patients and impairing the erythroid differentiation while stimulating the myelo-monocytic pathway [92].

#### Benzamides

Benzamides including entinostat (MS-275 or SNDX-275) and mocetinostat (MGCD103) are another class of molecules that demonstrate specificity for class I HDACs. Entinostat is a novel and orally available synthetic benzamide HDACis that preferentially inhibits HDAC1 but does not possess activity against HDAC6 [93]. Entinostat exerts dose-dependent effects on Jurkat T cell line: a p21-dependent growth arrest and differentiation at low drug concentrations and a marked induction of reactive oxygen species, mitochondrial damage, caspase activation, and apoptosis at higher concentrations [94]. Entinostat can increase the apoptosis in Jurkat T cell line and significantly improve the histone acetylation combined with 5-azacytidine [95]. The interaction between MS-275 and fludarabine in lymphoid and myeloid human leukemia cells was examined, and the results demonstrated that sequential treatment of Jurkat T cell line with MS-275 and fludarabine induces mitochondrial injury, caspase activation, and apoptosis [96]. However, entinostat and mocetinostat were mainly studied in acute myeloid leukemia, relapsed and refractory lymphoma, and chronic lymphocytic leukemia in the clinical trials.

## Conclusions

Epigenetic modifications are currently considered as fundamental hallmarks of human cancer, playing a pivotal role in tumorigenesis [97]. It is now widely accepted that epigenetic and genetic alterations collaborate in the development and maintenance of ALL [98]. Histone acetylation is potentially reversible, making them a valuable target in the fight against ALL. Therefore, many efforts have focused on designing and developing small-molecule inhibitors for reverting undesirable histone acetylation in ALL. Although ALL therapies based on HATs and HDACis seem promising, there are insufficient clinical data to establish whether this class of drugs will be efficacious in ALL treatment [98]. Knowledge related to the functional outcomes of histone acetylation in ALL has been indirectly derived through the use of inhibitors, signifying that many histone acetylation targets remain to be discovered and exploited. Despite significant advances, the future study still holds challenges including lack of predictive biomarkers, non-specificity of broad-spectrum histone acetylation drugs, and ambiguous mechanisms for therapeutic response/resistance [99].

The epigenetic modifications in ALL include DNA hypermethylation and histone modifications. Recently, it has become clear that the DNA and histone lysine methylation systems are highly interrelated and rely mechanistically on each other for normal chromatin function [100, 101]. It also showed that the combination of HDACis with other epigenetic agents, such as DNA methyltransferase inhibitors, can produce encouraging clinical and biologic activity [102]. Therefore, better outcomes could be achieved on the combination of HDACis with other epigenetic agents in the treatment of ALL.

In summary, histone acetylation is an extremely promising area of research that holds endless potential for future achievement in ALL. However, we have only seen the tip of the iceberg, and there are many crucial questions that remain to be answered.

## Abbreviations

ALL: acute lymphoblastic leukemia
HAT: histone acetyltransferase
HDAC: histone deacetylase
HDACis: HDAC inhibitors
SAHA: suberoylanilide hydroxamic acid
